# Adult Alveolar Soft Part Sarcoma of the Head and Neck: A Report of Two Cases and Literature Review

**DOI:** 10.1155/2014/597291

**Published:** 2014-12-23

**Authors:** Brandon T. Mullins, Trevor Hackman

**Affiliations:** ^1^School of Medicine, University of North Carolina School of Medicine, 1033 Bondurant Hall, Chapel Hill, NC 27514, USA; ^2^Division of Otolaryngology, University of North Carolina Hospitals, 101 Manning Drive, Chapel Hill, NC 27514, USA

## Abstract

*Background*. Alveolar soft part sarcomas (ASPS) of the head and neck are rare, aggressive soft-tissue malignancies. This study describes the clinical course and management of two patients presenting with ASPS in very rare head and neck locations, the larynx and parotid gland. *Methods*. We identified two patients presenting with ASPS of the head and neck and treated at the University of North Carolina. We compared our results to the literature from 1987 to 2013. *Results*. Patient ages at diagnosis were 27 and 39 with presenting symptoms of hoarseness and parotid swelling, respectively. Mean follow-up was 87 months. All patients received surgical resection and adjuvant radiotherapy. There were no recurrences or evidence of distant metastatic spread during the series. Disease-free survival time for the patients was 4 months and 168 months, respectively. *Conclusions*. Our study suggests that a combined-modality approach is important in the treatment of ASPS of the head and neck even in these rare locations. Continued research into new therapies is necessary to improve historically poor outcomes.

## 1. Introduction

Alveolar soft part sarcoma (ASPS) is a rare soft-tissue malignancy constituting less than 1% of soft-tissue sarcomas with only about 25% of those occurring in the head and neck [1–7]. In the head and neck region typical sites include the tongue and orbit, with other sites being very rare [2–4, 6–8]. It primarily affects adolescents and young adults (between the ages 15 and 35) and has a predilection for females over males [5–8]. Though they are relatively indolent in nature, due to their high rates of late-onset metastasis, they typically have a poor long-term prognosis [8, 9].

This IRB-approved retrospective case series describes two patients with ASPS of the head and neck treated at the University of North Carolina. Both patients present with very rare locations for head and neck ASPS (the larynx and parotid gland) with only a few known cases reported in the literature [2, 9–11]. They were diagnosed based on a thorough history and physical examination and pathologically confirmed through biopsy and histopathological analysis, with one case also including cytogenetic confirmation. For each case, wide-local surgical excision and adjuvant radiotherapy were the treatment of choice. Our goal is to add to the literature by reporting the clinical presentation, histopathologic and immunohistochemical findings, therapeutic considerations, and outcomes of two very rare locations for head and neck ASPS with a literature review.

## 2. Clinical Cases

### 2.1. Case 1

A 27-year-old male presented to our hospital with a 3-year history of hoarseness. He reported that the hoarseness had been worsening over the past few months and was newly associated with increased throat clearing and an occasional eating-associated cough. The past history was otherwise unremarkable.

Head and neck physical examination was unremarkable without evidence of masses, lesions, or cervical adenopathy. Endoscopy was then performed for further assessment. Flexible fiberoptic laryngoscopy revealed a left-sided submucosal mass of the false vocal cord obstructing the view of the underlying left true vocal cord. Normal mobility of the true vocal cords was noted and there was good glottis closure with pronation. The rest of the laryngeal exam was unremarkable.

**Figure 1:**
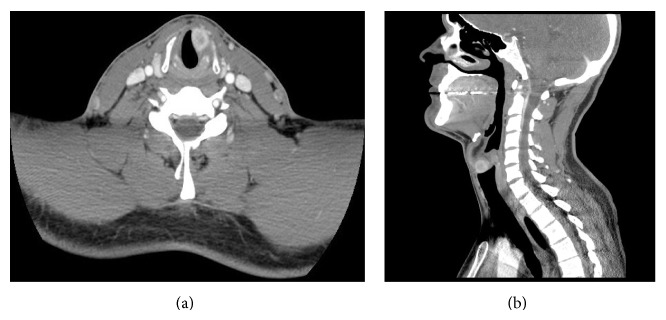
Axial (a) and sagittal (b) CT of the avidly enhancing mass in the anterior left vocal cord.

Computed tomography (CT) revealed a 1.5 × 1.2 × 1.5 cm avidly enhancing mass in the anterior left vocal cord (Figure 1). The lesion involved the true left vocal cord and extended superiorly into the left laryngeal ventricle to the level of the left false vocal cord. There was no involvement of the left epiglottic fold, but there was slight infraglottic extension. A chest radiograph was obtained for staging and ruled-out pulmonary metastases and mediastinal adenopathy. Biopsy was then performed under general anesthesia revealing a firm, yellow mass somewhat alveolar and grape-like in appearance.

Microscopic examination of the biopsy specimen revealed a multilobulated spindle cell lesion. Many of the lobules were composed of nests of large polygonal cells containing finely granular eosinophilic cytoplasm and separated by delicate vessels. Based on the structure, the differential diagnosis included ASPS, perivascular epithelioid cell tumor (PEComa), or possibly metastatic carcinoma.

Immunohistochemistry then revealed negative staining results for epithelial membrane antigen (EMA), cytokeratin AE1/AE3, Cam 5.2, S-100, HMB-45, Melan-A, and smooth muscle actin, with rare cells positive for desmin. TFE-3 then showed diffuse nuclear positivity. These results narrowed the differential to ASPS or PEComa. To confirm the diagnosis, FISH analysis was then performed to evaluate for TFE-3 gene rearrangement. A characteristic feature of ASPS is the unbalanced TFE-3 translocation; however, in this case, the translocation was determined to be balanced, even on repeated analysis. Given the observed morphology and gene rearrangement results in the limited biopsy sample, PEComa was the preliminary diagnosis.

The patient was consented for excision of the mass via an endoscopic left vertical hemilaryngectomy excising the left true and false vocal cord. Intraoperatively, all frozen section margins were reported to be clear of tumor; however, on final pathologic analysis, two of the frozen section margins contained residual tumor. Also, now evaluating a larger sample, pathology concluded that the characteristic microscopic appearance and the immunohistochemical results should override the molecular results and made the final diagnosis of ASPS.

Laser reexcision was then performed to manage the initial positive margins, resulting in negative margins, and it was determined that no residual tumor was present in the reexcised specimen. Considering the initial positive margins, the narrow surgical field, and the inability to obtain wide margins while sparing the larynx, the multidisciplinary tumor board decided to include adjuvant radiotherapy in the treatment plan to reduce the risk of local recurrence. The patient received radiotherapy (59.4 Gy) at an outside hospital closer to home due to additional family support. He responded well to the therapy, had no complications, and is disease-free 4 months after the completion of treatment.

### 2.2. Case 2

A 37-year-old female presented to our institution with a 2-year history of left cheek swelling. The swelling was located in the region of the parotid gland and was not associated with pain. The past history was otherwise unremarkable.

Head and neck physical examination revealed a 2 × 2 cm firm, nontender mass in the left parotid area. The mass did not appear to be fixed to the underlying tissues. No adenopathy was appreciated and the remainder of the exam was unremarkable. Flexible fiberoptic nasal laryngoscopy was then performed for further evaluation, also determined to be unremarkable. CT scan was previously obtained at an outside institution and was not available to report.

Fine-needle aspiration was then performed to evaluate the mass. The initial assessment reported the presence of oncocytic cells consistent with an oncocytoma. The patient was then consented for excision of the mass via a left superficial parotidectomy, which was completed with close surgical margins.

Microscopic examination of the excised mass revealed a 2.1 cm discrete, possibly encapsulated, tumor consisting of nests and sheets of large, moderately pleomorphic, and polygonal cells bounded by broad fibrous septae with abundant small vessels. The cells showed minimal nuclear pleomorphism, prominent single central nucleoli, and abundant clear to eosinophilic granular cytoplasm. Rare mitotic figures were also noted. The differential diagnosis included oncocytoma, myoepithelioma, melanoma, adult rhabdomyoma, and ASPS.

Immunohistochemistry then revealed focal reactivity to desmin with no reactivity for actin, S100, HMB45, keratin, synaptophysin, or chromogranin. This immunophenotype supported the diagnoses of adult type rhabdomyoma and alveolar soft part sarcoma and excluded the others. Periodic acid Schiff (PAS) staining was then performed revealing focal reactivity with some cells showing an intracytoplasmic crystalline material. It was then determined that the PAS positive structure combined with the desmin reactivity was most consistent with ASPS.

Since ASPS has a high metastatic rate, especially to the lung, chest CT was performed for staging and ruled out pulmonary metastases. Considering the close surgical margins, she then underwent adjuvant radiotherapy to a total dose of 63.4 Gy. There were no major complications from treatment. Since completion of radiotherapy she has had regular follow-up and has remained disease-free 168 months after the completion of her treatment.

## 3. Discussion

Alveolar soft part sarcomas are extremely rare soft-tissue sarcomas, typically occurring in the deep soft tissues of the lower extremities, and are especially rare in the head and neck [8, 9]. Clinically they are rather indolent in nature, with a slow clinical course, and usually present with functional impairment due to primary tumor location (i.e., hoarseness or dysphagia) or as a painless slowly enlarging mass, as seen in our cases (Table 1) [6, 8, 9]. However, due to their highly vascular nature, they also have a high rate of distant metastasis, with the lungs, brain, and bone being the most common sites [6, 8, 9]. Lung metastases are most common and are seen in 40–60% of cases [9, 12–14]. While hematogenous spread is the typical route, lymphatic metastasis is also seen in around 7–10% of cases [9, 15].

For the diagnosis of ASPS, imaging combined with analysis of the histologic, immunochemical, and molecular genetic features is beneficial. On imaging, the tumor demonstrates low attenuation on noncontrast CT and strong tumor enhancement with contrast administration. TI- and T2-weighted MRI images typically show higher signal intensity than muscle while demonstrating tubular areas of flow voids, representative of rapid blood flow within the tumor [16, 17]. Microscopically, ASPS is characterized by a pseudoalveolar appearance with clustered polygonal tumor cells, containing an abundant eosinophilic to clear granular cytoplasm, and separated by capillary-sized vascular channels and connective tissue [8, 12, 18]. On immunohistochemistry, the tumor cells are typically negative for epithelial markers (i.e., EMA and cytokeratins), chromogranin A, synaptophysin, S-100, HMB45, and Melan-A [8]. Desmin may be positive in around 50% of cases, and nonspecific markers such as neurone-specific enolase and vimentin may be positive in 30–50% of cases [8]. Most characteristically, ASPS exhibits cytoplasmic crystals containing PAS-positive and diastase-resistant material, which is seen in 80% of cases [9, 12, 15, 18]. In addition, on molecular genetic analysis, ASPS can be characterized by the der(17)t(X:17)(p11.2;p25) translocation. This unbalanced translocation between chromosomes 17q25 and Xp11.2 leads to the ASPL-TFE3 fusion gene, which activates the MET promoter and confirms the diagnosis [8, 11, 18–20]. The diagnostic features of the cases presented were mostly consistent with the literature. The tumor in case 1 demonstrated the expected enhancement on contrast imaging, microscopic examination in both cases revealed large polygonal cells with granular eosinophilic cytoplasm separated by small vessels, and immunohistochemical results in each case demonstrated either PAS-positive cytoplasmic crystalline material or TFE-3 nuclear positivity (representing the fusion gene) with most other markers staining negative. The major differing feature presented in case 1 was the balanced translocation between 17q25 and Xp11.2. However, though very rare, this balanced translocation has been reported a few times in the literature [21, 22]. The difficulty in reaching a final pathologic diagnosis in our cases, especially case 1, shows that careful and thorough diagnostic testing is essential for proper diagnosis of ASPS, especially in rarer anatomic locations.

Due to the rarity of ASPS of the head and neck, the optimal treatment plan has not been clearly elucidated. Currently, the main treatment of primary ASPS, like most soft-tissue sarcomas, is surgical removal using a wide-local excision with the goal of obtaining negative margins [6, 7, 9]. The attainment of negative margins after surgical resection has been shown to increase local control and survival rates. Due to the low rate of lymphatic spread, neck dissection is typically only utilized when palpable nodes are present rather than prophylactically [6, 9]. When metastatic lesions are present in the lung or brain, the current standard treatment is also surgical excision aiming for negative margins as it has been linked to increased median survival [18, 23, 24].

The role of radiotherapy has been controversial. Early studies reported no significant benefit with the addition of radiotherapy [24, 25]. For example, Lieberman et al., in a 102-patient review of ASPS at all sites from 1952 to 1987, reported that there was no survival advantage for patients treated with radiotherapy [24]. However, more recent studies by Sherman et al., Anderson et al., and Ogura et al., all, showed significant benefit in local control amongst their patients who received adjuvant radiotherapy after primary surgical removal [18, 26, 27]. In addition, Ogura et al. found radiotherapy benefit in treating patients with brain metastases due to ASPS [18]. Four of their patients with brain metastases were treated with gamma knife radiotherapy and all achieved satisfactory local control, with a median progression-free survival time of 12 months. They were all alive at last follow-up. Historically, the median survival time after brain metastases diagnosis from ASPS is 12 months [18]. There has been little study in this area, and more research should be conducted to evaluate the most efficacious treatment between gamma knife radiotherapy and surgical resection for ASPS brain metastases. The patients in our study were both treated with surgical resection and adjuvant radiotherapy which resulted in a good clinical result (Table 2). Both patients have remained recurrence-free, with progression-free survival times of 168 months and 4 months.

Conventional cytotoxic chemotherapy has been widely reported to have little benefit in the treatment of ASPS [13–15, 18, 24, 25, 27]. Many sources have cited the lack of efficacy for agents like doxorubicin, ifosfamide, dacarbazine, gemcitabine, vincristine, docetaxel, melphalan, and TNF-alpha [14, 18, 24, 25, 27–29]. Due to this, neither of the patients in our series underwent chemotherapy treatment. However, recently, more evidence has appeared for the use of antiangiogenic agents in the treatment of ASPS. Given the highly vascular nature of ASPS, antiangiogenic agents like bevacizumab, sunitinib malate, and cediranib have shown promise in small series and clinical trials for the treatment of primary and metastatic ASPS [18, 30–34]. Gardner et al. reported substantial single-agent effectiveness against ASPS in a 7-patient clinical trial [34]. Ogura et al. also noted significant tumor shrinkage with a progression-free survival period of 27 months in their patients treated with cediranib [18]. Based on early promising results such as these, continued research into the efficacy of these agents is needed to hopefully improve outcomes in ASPS. In addition, given that the ASPL-TFE3 fusion protein binds to the MET promoter and can lead to high rates of MET overexpression, molecularly targeted therapy against MET may also be a potential therapeutic target [35].

Despite current treatments, the long-term prognosis for ASPS has remained poor due to the high rate of metastatic disease. Lieberman et al. cited 2-year, 5-year, 10-year, and 20-year survival rates of 77%, 60%, 38%, and only 15%, respectively [24]. Similar 5-year and 10-year survival rates have also been reported in more recent studies [14, 18, 26]. Portera et al. reported that while the 5-year disease-free survival rate was 71% for patients presenting with localized disease, the rate dropped to 20% for patients presenting with metastases [14]. It has also been cited that median survival time decreased from a median of 11 years to 3 years when metastasis was present on presentation [9]. In recent studies, metastases on presentation have been reported in 48–65% of patients [14, 18, 20, 29], with posttreatment metastatic rates ranging from 19 to 24% [14, 18, 20]. The local recurrence rate has been similar, ranging from about 10 to 25% [14, 20, 24, 28, 36]. The rarity of ASPS in the head and neck region makes it difficult to determine prognostic factors for survival, as most studies are smaller clinical series. Though, when viewing studies based on ASPS of all sites, tumor size and AJCC stage have most readily been correlated with survival [14, 18, 24, 25, 29]. It is difficult to evaluate prognostic factor correlations in our small sample. Both patients had small tumors (less than 5 cm in size), presented at a low stage without metastasis, and received adjuvant radiotherapy despite margin status, and both patients are alive and have had good clinical outcomes.

In conclusion, our study reports two cases of head and neck ASPS in extremely rare locations, the larynx and parotid gland. When presenting in rare locations such as these, diagnosis can be difficult and analyses of the histopathologic, immunohistochemical, and molecular genetic features are beneficial for confirmation. Due to its rarity in the literature, the optimal treatment for ASPS has yet to be clearly elucidated. The most evidence seems to conclude that wide-local excision with adjuvant radiotherapy is the best current therapy, though long-term survival remains poor. Hopefully, continued research into new therapies such as antiangiogenic agents, as well as stringent follow-up for late-metastatic detection, will improve the poor outcomes of ASPS.

## Figures and Tables

**Table 1:** Clinical characteristics of the alveolar soft part sarcoma series.

Case	Age	Gender	Location	Tumor size	Symptoms
1	27	Male	Larynx	1.5 cm	Hoarseness
2	39	Female	Parotid gland	2.1 cm	Enlarging mass

**Table 2:** Treatment and outcomes of the alveolar soft part sarcoma series.

Case	Primary treatment	Margin status	Recurrence location	Time to recurrence	Disease-free survival
1	Surgery + adjuvant radiotherapy	Positive	N/A	N/A	4 months (Alive)
2	Surgery + adjuvant radiotherapy	Negative	N/A	N/A	168 months (Alive)
